# Prebiotics and probiotics in animal nutrition: implications for food safety, sustainability, and circular production systems

**DOI:** 10.1007/s42770-026-02033-4

**Published:** 2026-07-24

**Authors:** João Victor dos Anjos Almeida, Julia Memrava Cabrera, Mauro de Medeiros Oliveira, Ricardo Pinheiro de Souza Oliveira, Alessandro M. Varani

**Affiliations:** 1 School of Agricultural and Veterinarian Sciences, Sao Paulo State University (Unesp), Jaboticabal, SP Brazil; 2 School of Pharmaceutical Sciences, University of Sao Paulo, Sao Paulo, Brazil

**Keywords:** Feed additives, Food safety, Livestock nutrition, Microbiota modulation, One Health

## Abstract

The pursuit of sustainable and efficient animal production systems has intensified global interest in microbiota-targeted nutritional strategies. This review synthesizes current research on prebiotics and probiotics, selectively utilized microbial substrates and beneficial live microorganisms, respectively, that confer health benefits, across major livestock and aquaculture sectors, including poultry, swine, ruminants, fish, and shrimp. It examines the fundamental role of host-specific gut microbiomes in regulating health and productivity and elucidates how prebiotics, alone or in synergistic formulations with probiotics, modulate these microbial communities. Mechanisms of action involve enhanced nutrient digestibility, improved intestinal morphology and barrier integrity, selective stimulation of beneficial bacteria (e.g., *Lactobacillus*, *Bifidobacterium*), suppression of pathogens (e.g., *Escherichia coli*, *Salmonella*, *Vibrio*), and potentiation of innate and humoral immune responses. Consistent evidence demonstrates that microbiota-targeted interventions improve key zootechnical indicators, including weight gain, feed conversion ratio, and carcass quality, while enhancing animal welfare and resilience to environmental and physiological stressors. A pivotal finding is their ability to reduce reliance on antimicrobial agents, addressing antibiotic resistance and aligning with consumer demand for ethical and natural production systems. Furthermore, this review highlights the valorisation of agro-industrial by-products (e.g., fruit pomace, cereal brans) and novel substrates such as *Hermetia illucens* (black soldier fly) as functional, cost-effective sources that embed animal nutrition within a circular bioeconomy framework. By integrating multidisciplinary findings from terrestrial and aquatic species, this review concludes that microbiota-based functional nutrition is indispensable for advancing next-generation sustainable animal production, uniting productivity, environmental stewardship, and animal welfare while supporting safe, ethical, and climate-resilient food systems worldwide.

**Graphical abstract:**

Probiotic and prebiotic integration in swine, poultry, cattle, and aquaculture systems. Supports animal health, feed efficiency, and sustainable by-product use, aligning with SDG 2 and SDG 12. Repurposing agro-industrial residues as novel prebiotics closes the resource loop, reinforcing the circular economy.

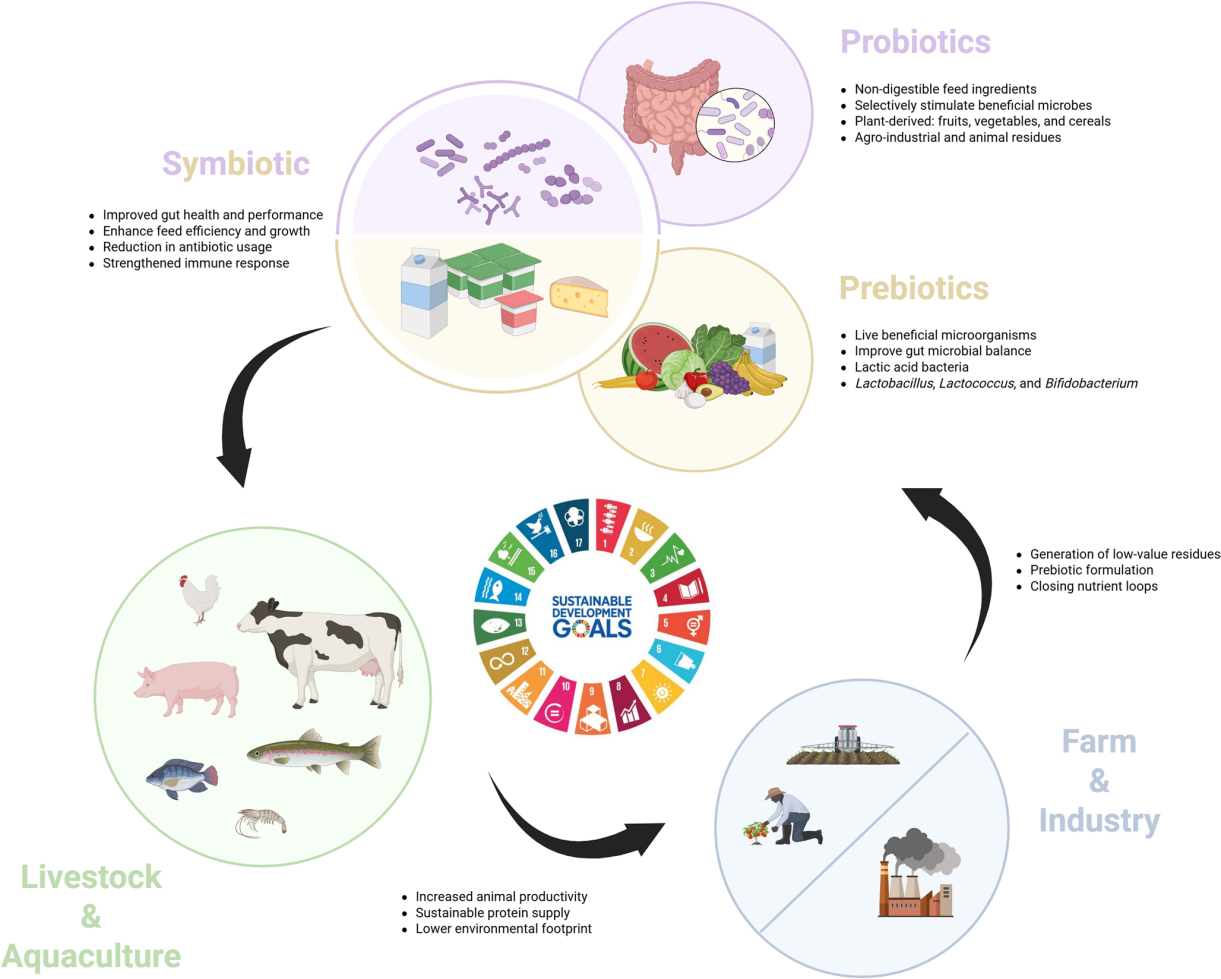

## Introduction

**Fig. 1:**
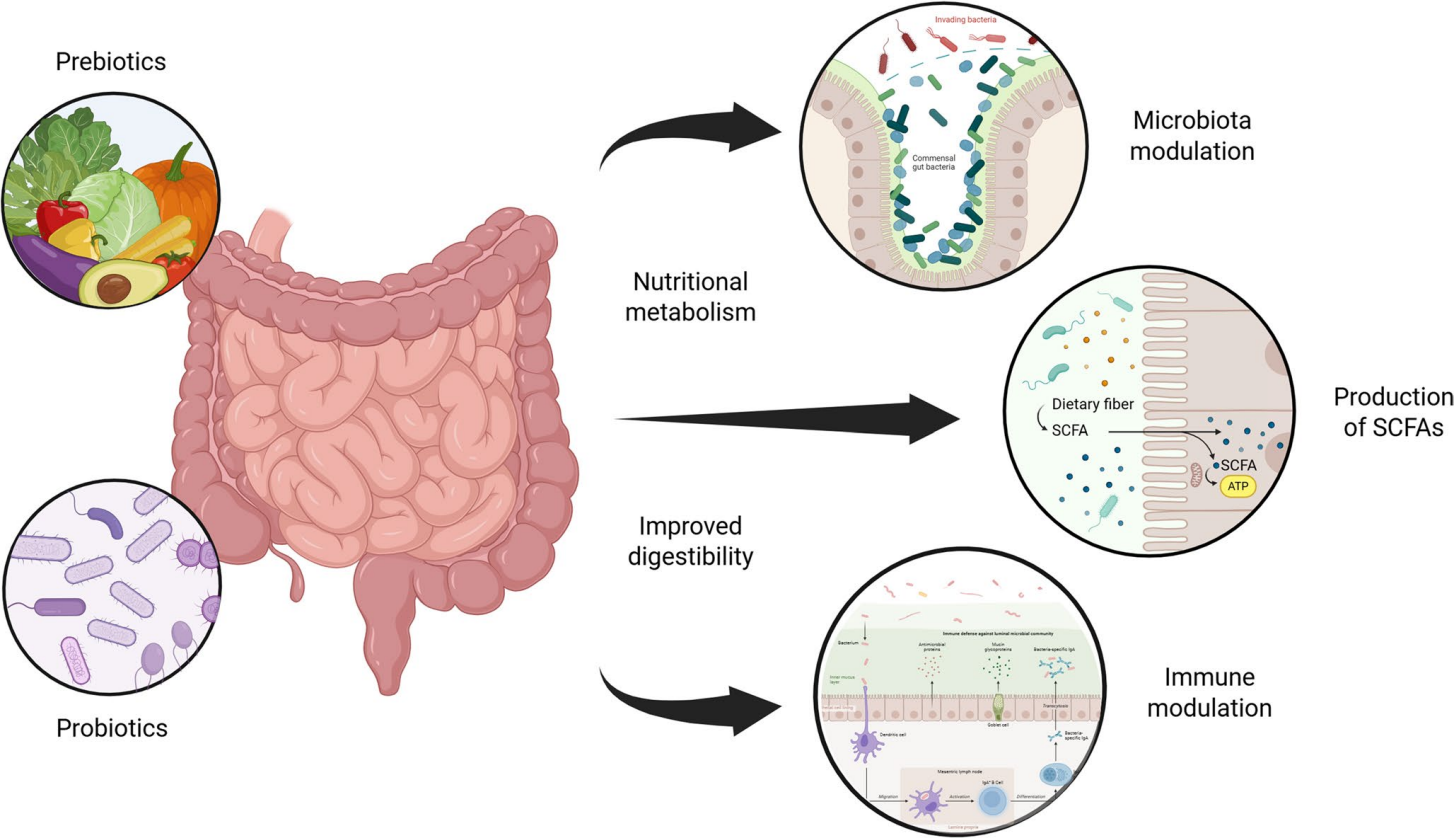
Schematic representation of the mechanisms by which prebiotics and probiotics modulate intestinal health. Prebiotics derived from dietary fibers and probiotics from beneficial microorganisms act synergistically to improve nutritional metabolism, enhance digestibility, and promote gut homeostasis

The global food system faces increasing pressure to produce animal protein efficiently, safely, and sustainably, while minimizing the environmental footprint of intensive production. Antimicrobial resistance, greenhouse gas emissions, and growing consumer demand for antibiotic-free and welfare-oriented products have catalysed a change in basic assumptions toward sustainable, microbiota-targeted nutrition. Within this context, prebiotics and probiotics have emerged as key biotechnological tools to enhance gut health, improve feed efficiency, and strengthen host resilience without relying on antimicrobials (Fig. 1). These functional feed additives not only modulate the gut microbiota but also contribute to food safety and public health by lowering pathogen prevalence, improving nutrient utilization, and reducing environmental emissions. 

In this framework, microbiome-informed nutritional strategies provide a bridge between food safety, sustainability, and animal welfare. The concepts of prebiotics and probiotics, defined by the International Scientific Association for Probiotics and Prebiotics (ISAPP) as “substrates that are selectively utilised by host microorganisms conferring a health benefit” and “live microorganisms that, when administered in adequate amounts, confer a health benefit on the host,” respectively, are now central to developing sustainable animal production systems [1]. Their combined, synergistic use integrates advances in microbial ecology, feed biotechnology, and circular economy principles, aligning with the One Health framework and the United Nations Sustainable Development Goals (SDGs).

While probiotics introduce beneficial microbes into the gastrointestinal tract, prebiotics function as selective substrates that stimulate their growth or activity, thereby enhancing nutrient absorption, short-chain fatty acid (SCFA) production, mucosal integrity, and immune modulation [1–3]. This synbiotic interaction, extensively studied in human health [4], is increasingly explored in animal systems. In livestock species such as poultry, swine, cattle, and fish, prebiotics have been associated with improvements in weight gain, disease resistance, and physiological resilience to stress [5, 6].

Among the most widely studied probiotics in both human and animal health are lactic acid bacteria (LAB), including *Lactobacillus*, *Lactococcus*, and *Bifidobacterium*. These organisms are recognised for their antimicrobial activity, metabolic versatility, and applications in food preservation, fermentation, and safety [7, 8]. Their combination with prebiotics enhances microbiota composition and functional benefits in the host, providing synergistic effects on gut health and immunity [9].

Furthermore, incorporating prebiotics into animal diets supports host health while promoting the biotechnological valorisation of agro-industrial by-products, reinforcing circular economy principles and food safety [2, 10]. Although research on prebiotics has historically focused on human applications, their integration into animal production systems represents a promising approach for advancing sustainable, efficient, and environmentally responsible livestock systems, in line with the goals of the United Nations 2030 Agenda.

Building upon these principles, the following sections synthesize current advances in nutritional biotechnology across major animal production systems. By integrating microbiome-driven strategies into poultry, swine, cattle, aquaculture, and shrimp farming, as well as exploring novel bioactive sources such as the black soldier fly, this review provides a comparative overview of how prebiotics and probiotics can optimize intestinal health, immune function, and productivity. Emphasis is placed on their synergistic roles in reducing antibiotic dependence, enhancing feed efficiency, and aligning livestock and aquaculture production with sustainability and food safety goals. Together, these insights illustrate how microbiota-targeted nutrition serves as a unifying framework bridging molecular innovation, animal welfare, and environmental resilience across the global food system.

This article is a narrative review. Relevant literature was identified through searches of PubMed, Scopus, and Web of Science, complemented by targeted searches of Google Scholar and FAO/market-report databases for production and economic statistics, covering studies published between 2004 and 2026, with emphasis on the last five years. Search terms combined the concepts “prebiotic”, “probiotic”, “synbiotic”, and “gut microbiota” with species- or system-specific terms (e.g., “poultry”, “broiler”, “swine”, “cattle”, “ruminant”, “aquaculture”, “tilapia”, “rainbow trout”, “shrimp”, “Hermetia illucens”) and with outcome-related terms (e.g., “growth performance”, “immune response”, “intestinal morphology”, “feed conversion”, “circular economy”). Studies were considered for inclusion when they reported original experimental or field data on prebiotic, probiotic, or synbiotic interventions in livestock or aquaculture species and addressed at least one of the following outcome domains: zootechnical performance, intestinal microbiota composition, immune or antioxidant response, intestinal morphology, or environmental/circular-economy outcomes. Review articles, conference abstracts without peer-reviewed full text, and studies in non-production animal models were generally excluded, except where cited for background, mechanistic, or definitional purposes (e.g., refs [1–9]). Given the breadth of the subject and the diversity of production systems and outcome measures covered, a narrative synthesis approach was adopted rather than a systematic or scoping review framework, allowing for an integrative, mechanism-oriented comparison across species; accordingly, PRISMA/PRISMA-ScR reporting, which presupposes a pre-defined and quantitatively documented screening protocol, was not applied.

## Nutrition biotechnology in poultry production: impacts on intestinal health, immunity and productivity

Global poultry production, driven primarily by chicken meat, reached 150.1 million tonnes in 2024, fuelled by growing consumer demand and lower production costs, with Brazil consolidating its position as the world’s leading exporter [11, 12]. The four largest producers, China, United States, Brazil, and European Union, collectively accounted for most of the global output, reflecting their advanced feed industries, strong export capacity, and well-established poultry sectors [11]. On the consumption side, United States, China, Brazil, and Mexico remained the largest markets, while countries in Southeast Asia and the Middle East, including Japan and the United Arab Emirates, relied increasingly on imports to meet domestic demand [11]. This globalized structure highlights the interdependence of production hubs and consumer regions, emphasizing the need for efficient, resilient, and sustainable production chains.

Within this context, nutritional biotechnology has gained prominence as a key lever to enhance productivity and sustainability. The combined use of probiotics and prebiotics, often referred to as synbiotics, has emerged as a promising approach to improve intestinal health, boost growth performance, and reduce dependence on antibiotics, while simultaneously enabling the valorisation of functional feed ingredients and agro-industrial by-products [13].

### Microbiome profile and taxonomic overview in poultry

The chicken gut microbiome constitutes a dynamic and multifactorial ecosystem shaped by host genetics, production systems, and geographical conditions, with profound implications for animal health, immunity, and productivity [14]. At the phylum level, Firmicutes and Bacteroidota dominate the intestinal microbiota [15, 16]. Within Firmicutes, the class Bacilli encompasses key LAB, including *Ligilactobacillus salivarius*, *Lactobacillus ingluviei*, and *Lactobacillus aviarius*, which play essential roles in fermentation, immune modulation, and maintenance of gut homeostasis [16].

Production systems exert a marked influence on microbial composition: Bacteroidota predominate under extensive systems, whereas Firmicutes are more abundant in intensive production, a pattern often associated with reduced microbial diversity and loss of host-adapted taxa [17]. Geographical variation also contributes to microbiota structure; Chinese flocks tend to show greater abundance of antimicrobial resistance genes, while European samples display higher microbial diversity and enrichment of plasmid-associated taxa [15]. These patterns reflect complex interactions among diet composition, environmental variables (e.g., climate and temperature), management practices, and host genetics, which together determine gut community assembly.

Among these factors, host genetic background exerts particularly strong selective pressure on microbial assemblages. For instance, Chinese Silkie chickens exhibit enrichment of *Mucispirillum schaedleri* and *L. aviarius*, whereas Henan Gamecocks harbour higher levels of Vibrio and Proteobacteria [18]. Obese chickens display increased abundance of *Erysipelatoclostridium ramosum* and *Blautia*, taxa associated with carbohydrate metabolism and lipid deposition [19], suggesting that microbiota-mediated fat regulation in poultry operates partially independently of host genotype [20]. However, such taxonomic shifts do not always translate into consistent performance outcomes, as their effects depend on microbial interactions and environmental context.

The functional potential of specific taxa underscores the flexibility of the chicken microbiome as a genetically influenced yet environmentally responsive system. Probiotic supplementation, using *Lactobacillus* [21], *Bifidobacterium* [22], or mixed formulations [23], can remodel the gut microbiota, enrich beneficial taxa, and improve intestinal morphology, even when growth performance remains unchanged. Notably, *Lactobacillus plantarum* supplementation suppresses *Salmonella* infection in broilers [24], exemplifying the direct and indirect benefits of microbiota modulation for health and productivity in poultry production.

### Biotechnological potential of probiotics in poultry production

Probiotics exert multifaceted benefits on chicken health, performance, and intestinal integrity, acting as key biotechnological tools in sustainable poultry production. Supplementation with *Enterococcus lactis* TRM58998 in laying quails improved egg production, antioxidant status, and follicle development, suggesting systemic effects beyond gut colonization [25]. Similarly, *Enterococcus faecium* NCIMB 11181 alleviated necrotic enteritis-induced intestinal damage by upregulating tight-junction proteins and modulating immune responses [26].

In rustic chickens, *Lactobacillus acidophilus* D2/CSL enhanced villus height and *Lactobacillus* abundance, improving intestinal morphology and absorptive efficiency [27]. In industrial broilers, *L. salivarius* supplementation promoted growth and antioxidant capacity through gut–brain axis modulation, with contributions from *Megamonas*, *Ruminococcus*, and *Alistipes* [28]. Fermented feeds combining *L. salivarius* and *Lactobacillus crispatus* improved feed conversion ratio, egg quality, and microbial diversity by enriching *Ruminococcaceae* while reducing *Rikenellaceae* and *Methanobacteriaceae* [29].

Probiotic consortia integrating *Bacillus subtilis* and *E. faecium* with autolyzed yeast, mannan-oligosaccharides (MOS), and β-glucans have improved carcass yield and production efficiency while reducing antibiotic dependence [30]. These outcomes illustrate how prebiotics can complement probiotics by selectively stimulating beneficial microbes, enhancing gut function, and supporting overall performance.

This complementarity is further demonstrated by commercial synbiotic formulations that combine specific prebiotics and probiotics to enhance productivity and resilience. For instance, Sohail *et al*. [31] reported improved performance using 0.5% MOS (Safmannan^®^; Lesaffre Feed Additive, Marquette-Lez-Lille, France) combined with Protexin^®^ (Probiotics International Ltd., Somerset, UK), while Salem and El-Dayem [32] observed similar benefits with *TechnoMos*^®^ (Biochem Zusatzstoffe Handels- und Produktionsgesellschaft mbH, Lohne, Germany) paired with *Bio-Plus 2B*^®^ (Chr. Hansen A/S, Hoersholm, Denmark). Together, these findings underscore the emerging industrial shift toward synbiotic strategies to optimize poultry production while advancing food safety, antibiotic stewardship, and sustainability goals.

### Impact of prebiotic associations on poultry nutrition

Prebiotic supplementation enhances intestinal health, immune responses, and overall performance in poultry while contributing to circular economy practices (Table 1). The use of these bioactive organic materials promotes more efficient nutrition, reflected in improved final body weight [33, 34] and feed conversion ratio (34,35,36,37,39], by promoting resource circulation within production systems and reducing waste, thereby supporting animal health and productivity.

**Table 1:** Effects of different prebiotics on digestibility, zootechnical performance, immune response modulation, intestinal microbiota, and morphology in poultry. Values are expressed as a percentage (%) increase or reduction compared to the control group, or as an absolute change, as described in the original studies

Prebiotics	Digestibility and metabolic improvements	Growth and production performance	Immune enhancement	Microbiota modulation	Morphology	Studies
Astragalus, shiitake, and tremella	-	ADG + 17.6–26.5 g/day	-	↑ Bifidobacteria and Lactobacilli; ↓ *E. coli* and *Bacteroides* spp.	-	[35]
Fermented grape seed meal	CP + 2.2%; NDF − 6.82%; ADF − 8.23%	ADG 36.43–42.59 g/day; FCR − 11.7%; Abdominal fat − 29.6%	-	↑ Firmicutes; ↓ Bacteroidetes	-	[36]
Fermented soybean meal	CP + 2.46%	BW + 103 g; FCR − 5.9%	H:L -15–30%	↑ LAB; ↓ Coliforms	VH duodenum + 8%; VH/CD + 10%	[37]
Fermented yuzu by-products	-	BW + 171%; ADG + 9.9%	IgM + 49%	↓ *E. coli*	-	[33]
Insoluble fibre and whole wheat	Ileal starch digestibility + 9%	-	-	-	Gizzard weight + 39%; Gizzard content + 183%	[38]
Low-phytate peas	Liver iron + 27%; Muscle glycogen + 67%	BW + 16%	-	↑ *Bifidobacterium* and *Lactobacillus*	-	[34]
Seaweed blend	-	BW + 183 g; FCR − 2.3%	-	↑ Firmicutes; ↓ Actinobacteria and Proteobacteria	-	[39]
*Spirulina platensis* and Garlic powder	MDA − 31%; TAC + 51%; Cholesterol − 23%; LDL − 39%; TG -10%	BW + 13.5%; FCR − 10%	IgM + 87%; IgY + 43.5%; NDV + 25%; IBD + 49%	↑ *Lactobacillus*; ↓ Coliforms	VH + 3.2; VH: CD + 11.5	[40]
Unripe banana flour	-	BW + 175 g; FCR − 15.8%	-	-	-	[41]

*ADG* Average Daily Gain, *ADF* Acid Detergent Fiber, *BW* Body Weight, *CD* Crypt Depth, *CP* Crude Protein, *FCR* Feed Conversion Ratio, *H:L* Heterophil/Lymphocyte ratio, *IBD* Antibodies Against Infectious Bursal Disease, *LDL-C* Low-Density Lipoprotein Cholesterol, *MDA* Malondialdehyde, *NDF* Neutral Detergent Fiber, *NDV* Antibodies Against Newcastle Disease Virus, *TAC* Total Antioxidant Capacity, *TG* Triglycerides, *VH* Villus Height, *VH/CD* Villus Height/Crypt Depth Ratio

These physiological improvements are strongly associated with changes in the gut microbial ecosystem. Fructooligosaccharide (FOS) supplementation, for example, increased Lactobacillaceae abundance and reduced Lachnospiraceae [42], while β-glucan-enriched diets favoured *Faecalibacterium* and *Bacteroides*, enhancing intestinal stability [43]. Fermented by-products such as grape seed cake [36] and plant-derived substrates [44, 45] also supported proliferation of beneficial taxa, reinforcing the concept that properly processed plant matrices can yield synergistic microbial benefits. Together, these microbial shifts underpin the observed improvements in performance and gut functionality, bridging nutritional inputs with microbial metabolism.

Beyond microbiological and digestive functions, prebiotic interventions generate broader environmental and sustainability benefits. Studies report reduced ammonia emissions and improved litter quality [46], along with the valorisation of agro-industrial co-products such as citrus fibers [36, 44, 45] and wheat bran arabinoxylans [46] as functional, low-cost feed ingredients. These outcomes demonstrate how nutritional innovation can simultaneously improve productivity and ecological efficiency, reducing reliance on conventional feedstocks such as corn and soy.

At the physiological level, many prebiotics exert potent immunomodulatory and barrier-enhancing effects. Supplementation with inulin [47], FOS [48], or yeast-derived prebiotics [49] increased immunoglobulin levels (IgA, IgM, IgG) and downregulated pro-inflammatory cytokines, Interleukin-1 β (IL-1β), and Interferon-γ (IFN-γ). MOS improved villus height and the villus-to-crypt ratio even under *Salmonella* challenge [50], whereas xylooligosaccharides (XOS) [51] and macroalgae-derived prebiotics [52] enhanced SCFA production, energy metabolism, and intestinal barrier function. These findings reveal how prebiotics orchestrate microbial, immune, and metabolic pathways to reinforce host resilience.

Collectively, the synergy between functional nutrition, animal health, and waste valorisation enhances both the economic and ecological viability of poultry production. By improving survivability, carcass yield, and feed efficiency while lowering antibiotic use, prebiotics emerge as key biotechnological tools for next-generation poultry systems that integrate productivity, profitability, and ethical responsibility.

## Nutrition biotechnology in swine production: impacts on intestinal health, immunity and productivity

Global swine production reached 125.1 million tonnes in 2024, remaining stable compared with the previous year [11]. The leading producers, China, European Union, United States, Brazil, Russian Federation, and Vietnam, collectively accounted for most of the world’s output. Declines in China, the Philippines, Canada, and Japan were offset by gains in the European Union, United States, Russia, and Vietnam. Brazil, the fourth-largest producer, recorded a modest increase driven by robust international demand and expanding domestic consumption [11]. On the consumption side, China, United States, European Union, Brazil, and Mexico remained the largest markets, while import demand grew in the Philippines, the Republic of Korea, and Japan. Exports from the United States and Brazil continued to rise, partially compensating for lower shipments from the European Union and underscoring the adaptive and globalized nature of the swine industry [11].

In this highly competitive and interconnected production landscape, optimizing health, feed efficiency, and resilience has become essential. Given the critical role of the gut microbiota in the digestion of omnivorous diets [53], nutritional biotechnology offers powerful tools to achieve these goals. Strategic interventions, such as LAB-based probiotics to strengthen beneficial microbial populations and fiber-rich prebiotics to nourish them, can synergistically enhance feed conversion, improve intestinal health, and bolster immune competence while reducing dependence on synthetic additives and antibiotic growth promoters.

### Microbiome profile and taxonomic overview in swine

The swine gut microbiota is composed of Firmicutes, Bacteroidetes, and Proteobacteria, with core genera including *Bacteroides*, *Prevotella*, *Lactobacillus*, *Clostridium sensu stricto*, *Fusobacterium*, *Ruminococcus*, *Escherichia*, and *Blautia*, consistently identified in more than 90% of healthy individuals [54]. Age-related compositional shifts have been well documented: neonates exhibit higher proportions of *Bacteroides*, *Escherichia*, and *Lactobacillus*, while post-weaning pigs show increased abundance of fiber-degrading taxa such as *Prevotella* and *Anaerovibrio* [54]. These successional dynamics reflect microbial adaptation to dietary transitions from milk to solid feed and underline the importance of early-life microbial establishment for long-term intestinal stability.

Production systems exert a strong influence on microbial structure and diversity. In intensive indoor systems, which dominate global production, the gut microbiota retains a consistent core dominated by Firmicutes and Bacteroidetes, with *Lactobacillus* prevailing in the jejunum and greater microbial diversity in the cecum and colon. Shifts in the Firmicutes/Bacteroidetes ratio have been correlated with productive performance [55]. Conversely, extensive or traditional systems, such as those of Tibetan pigs, exhibit greater microbial diversity and enrichment of Fibrobacterota and Elusimicrobiota, whereas indoor systems favour *Escherichia*, Lactobacillaceae, and antimicrobial resistance gene enrichment [56].

Comparative studies between wild and domestic pigs further highlight the interplay between environment and microbial ecology. Wild boars harbour higher abundances of Elusimicrobiota, Verrucomicrobiota, and Cyanobacteria, yet share core taxa, particularly *Prevotella* and *Lactobacillus*, with domesticated populations [57]. This overlap indicates a conserved microbial foundation shaped by host phylogeny, modulated by diet and management conditions.

Expanded metagenomic catalogues have identified the consistent presence of *Limosilactobacillus reuteri*, *Lactobacillus johnsonii*, *Lactobacillus amylovorus*, *Escherichia coli*, *Prevotella copri*, and *Streptococcus suis* across intestinal compartments [58]. These findings underscore the functional plasticity of the swine gut microbiota and its central role as a target for microbiota-driven nutritional interventions aimed at improving feed efficiency, immune competence, and overall productivity.

### Biotechnological potential of probiotics in swine production

Supplementation with *L. plantarum* JDFM LP11 in weaned piglets significantly increased microbial diversity and enriched beneficial taxa such as Ruminococcaceae, while reducing pro-inflammatory groups [59]. These changes were associated with improved mucosal integrity, elevated serum IgG levels, and activation of microbial pathways linked to butyrate production and branched-chain amino acid biosynthesis, indicating enhanced metabolic and immune function [59].

Similarly, *L. reuteri* ZLR003 supplementation enriched *Lachnospiraceae*, key SCFA producers, and was accompanied by higher serum IFN-γ levels [60]. Concurrently, reductions in *Terrisporobacter* were linked to increased concentrations of anti-inflammatory cytokines IL-10 and IL-4, as well as immunoglobulins IgM, IgG, and IgA. These outcomes underscore the dual functional role of LAB in the gastrointestinal tract: acting as commensal stabilizers that maintain microbial homeostasis and as active immunomodulators reinforcing the gut–immune axis and systemic host defence [60].

Other LAB strains, including *Pediococcus pentosaceus* SMFM2016-WK1, have also demonstrated notable probiotic potential, improving growth performance and microbiota composition in weaned pigs [61]. Co-fermented additives combining *L. plantarum* and *Pediococcus acidilactici* enriched SCFA-producing populations, translating into measurable improvements in nutrient utilization and productivity. Likewise, supplementation with *E. faecium*, either live or heat-killed, enhanced both growth and microbial stability, reinforcing the industrial viability and safety of LAB-based feed additives [62].

Additionally, certain LAB strains such as *L. plantarum* B2984 [63] and *Bifidobacterium longum* subsp. *infantis* CECT 7210 [64] possess the capacity to degrade prebiotic substrates, including FOS, inulin, and lactulose. This metabolic flexibility enhances colonization efficiency and promotes the synthesis of beneficial metabolites, amplifying intestinal health effects.

In commercial applications, these mechanisms underpin the success of probiotics such as Cylactin^®^ LBC (DSM Nutritional Products, Kaiseraugst, Switzerland) and Bio-plus2B^®^ (Chr. Hansen A/S, Hoersholm, Denmark) when used in combination with prebiotics like FOS. Similar outcomes have been achieved with synbiotic formulations such as EnzaPro^®^ (BioResource International Inc., Durham, NC, USA), which optimize gut functionality and productive performance [65, 66]. Collectively, these advances reflect the growing biotechnological and economic relevance of LAB-based synbiotic strategies in swine nutrition, paving the way for the development of next-generation, patentable feed technologies that integrate microbiome science with precision nutrition.

### Impact of prebiotic associations on swine nutrition

Prebiotic supplementation has consistently improved swine productivity, gut health, and welfare by selectively stimulating beneficial taxa while suppressing pathogenic populations (Table 2). Functional fibers such as cocoa hulls [67], chicory [68], grape pomace [69], and fermented bamboo fiber [70] have been shown to enrich *Faecalibacterium prausnitzii*, *Bacteroides*, *Prevotella*, *Roseburia*, *Coprococcus*, and *Alistipes*, while reducing *Clostridium histolyticum*, *Fusobacterium*, and *Sutterella*. These microbial shifts support nutrient utilization, intestinal stability, and overall host resilience.

**Table 2:** Effects of different prebiotics on digestibility, zootechnical performance, immune response modulation, intestinal microbiota, and morphology in swine. Values are expressed as a percentage (%) increase or reduction compared to the control group, or as an absolute change, as described in the original studies

Prebiotics	Digestibility and metabolic improvements	Growth and production performance	Immune enhancement	Microbiota modulation	Morphology	Studies
*Chlorella vulgaris*	PB -4.8%; ADFI + 11,4%	Stable ADG (~ 535–581 g/day, NS)	-	↑ *Lactobacillus*, *Oscillospira*, *Colidextribacter*, and *Helicobacter*; ↓ *Ruminococcus*, *Treponema*, and *Mitsuokella*	VH + 25%; VH/CD 32.8–41%	[71]
Fermented ginseng residues	↑ Glucose, TP and GLO; ↓ Creatinine, cholesterol, triglycerides, faecal gas (NH₃: 13.49 to 11.67 ppm; H₂S: 1.25 to 1.18 ppm; CO₂: 0.84 to 0.77 ppm)	BW + 6.5–8%; ↑ ADG; ↓ Diarrhoea 12.37% to 2.62%	↑ IgA, IgG, IL-4 SOD + 25.9% GSH + 20.8–73.4% MDA − 62.9%	↑ Firmicutes, *Bacteroides*, *Bifidobacterium*, and *Lactobacillus*; ↓ *E. coli*	-	[72]
High-fiber diet (25% TDF, 5% fast-fermentable fiber)	GE + 3.3%; CP + 0.7%	LWG + 5.6%	IL-8 -36%	↑ Prevotellaceae, Ruminococcaceae, Lachnospiraceae, and Fibrobacteraceae	-	[73]
Inulin (0.5%)	↑ SCFAs: Acetate + 46% and Butyrate + 52%	-	↑ IL-10; Decreases IL-6 (ileum − 30%; cecum − 19%); TNF-α (ileum − 17%; cecum − 20%)	↑ Bacteroidetes, *Lactobacillus*, and *Bacteroides*; ↓ Proteobacteria	VH + 11%; ↑ ZO-1 expression; ↓ Epithelial apoptosis	[74]
Mango pulp (15%) and Pectin	↑ SCFAs (Manga + 44% and Pectin + 68%); ↓ NH₄⁺ (Mango and Pectin)	-	-	↑ Diversity (Shannon in the cecum: Mango 6.35 vs. Pectin 4.45)	-	[75]
Wheat bran (5%)	-	ADG + 6.8%; FCR − 5.7–7.5%	-	↑ Fibrobacter; ↓ Prevotellaceae	-	[76]
Whole-plant silage maize (10%)	ADF + 4.92%; ↑ SCFAs	-	-	↑ *Alloprevotella* (+ 30.2%) and *Terrisporobacter* (+ 44.2%)	-	[77]

*ADF* Acid Detergent Fiber, *ADF I* Acid Detergent Fiber Intake, *ADG* Average Daily Gain, *AP* Apple Pomace, *BW* Body Weight, *CD* Crypt Depth, *CP* Crude Protein, *FCR* Feed Conversion Ratio, *GE* Gross Energy, *GLO* Globulin, *GSH* Reduced Glutathione, *LWG* Live Weight Gain, *MDA* Malondialdehyde, *PB* Crude Protein, *SBP* Sugar Beet Pulp, *SH* Soy Hulls, *SOD* Superoxide Dismutase, *TNF-α* Tumor Necrosis Factor alpha, *TP* Total Protein, *VH* Villus Height, *VH/CD* Villus Height / Crypt Depth ratio, *ZO-1* Zonula Occludens-1

Prebiotics also enhance resistance to enteric pathogens. For example, FOS supplementation reduced diarrhoea incidence in weaned piglets [78], whereas chicory decreased *Campylobacter* shedding [79]. Similarly, microfibrillated cellulose promoted the growth of butyrate-producing genera such as *Ruminococcus* and *Roseburia*, thereby reinforcing intestinal barrier function and mucosal integrity [80]. Collectively, these effects indicate that prebiotics reshape the gut microbiome toward SCFA-producing communities, creating a more resilient intestinal ecosystem. This dual effect, stimulating beneficial microbes while suppressing pathogens, underpins the improvements observed in gut morphology, immune parameters, and performance. Such coordinated microbial modulation is particularly valuable during early life and under high-density rearing conditions, where gut stability determines long-term productivity and disease resistance.

Among the immunomodulatory effects, isomaltooligosaccharide supplementation increased colostral and serum levels of IgA, IgG, and IgM in sows, supporting passive immunity transfer [81]. Enhanced intestinal morphology, characterized by increased villus height and villus-to-crypt ratios, has been reported after dietary inclusion of citrus pulp and inulin [82] or *Chlorella vulgaris* [71], leading to greater absorptive efficiency.

Performance gains include higher average daily gain, reduced feed conversion ratios, and lower oxidative stress markers such as malondialdehyde following fermented fiber supplementation [83, 84]. From an environmental standpoint, prebiotic-rich formulations have been associated with reduced faecal gas emissions and valorisation of agro-industrial residues such as sugar beet pulp, apple pomace, and soybean hulls [85], contributing to circular and low-emission livestock production.

Overall, these findings highlight the multifunctional potential of prebiotics, and their synergistic use with probiotics, to optimize gut health, modulate immunity, and enhance productive efficiency. By linking animal welfare and environmental responsibility, prebiotics emerge as integral tools for building sustainable and microbiota-informed swine production systems.

## Nutrition biotechnology in cattle production: impacts on intestinal health, immunity and productivity

Global bovine meat production reached 78.7 million tonnes in 2024, with Brazil, Australia, China, and European Union leading output, and emerging producers such as India showing notable increases [11]. In contrast, traditional exporters like Argentina and New Zealand experienced slight declines due to regional constraints and market fluctuations. Global beef trade expanded to 13.1 million tonnes, representing a 9.8% increase over the previous year, driven primarily by rising import demand in the United States, China, and Iran, while imports declined in markets such as Indonesia and South Korea, reflecting evolving consumption patterns and trade dynamics [11].

Within this competitive and rapidly evolving sector, nutritional biotechnology has emerged as a key enabler of sustainable intensification. Strategies that integrate LAB, fiber-rich substrates, and agro-industrial co-products are increasingly applied to enhance feed efficiency, modulate the ruminal microbiota, and mitigate methane emissions. These approaches contribute to climate-smart ruminant production systems that improve animal performance and health while promoting environmental sustainability. In particular, the valorisation of agricultural residues as functional feed ingredients aligns bovine nutrition with circular economy principles, offering dual benefits of productivity enhancement and greenhouse gas reduction.

### Microbiome profile and taxonomic overview in cattle

The bovine gastrointestinal microbiota, particularly that of the rumen, is dominated by Bacteroidetes and Firmicutes, followed by Proteobacteria, Spirochaetes, Fibrobacteres, and methanogenic archaea such as *Methanobrevibacter* (phylum Euryarchaeota) [86, 87]. These microbial communities are central to fiber degradation, SCFA production, and nutrient supply to the host [86, 88].

Marked differences in diet composition between intensive and extensive production systems strongly influence rumen microbial ecology. Extensive, pasture-based systems rich in structural carbohydrates favour cellulolytic taxa such as Lachnospiraceae, Eubacteriaceae, and Ruminococcaceae, which promote acetate and methane production [89]. In contrast, intensive systems characterized by grain- and starch-rich diets harbour higher abundances of *Bacteroidetes*, Selenomonadaceae, and Acidaminococcaceae, taxa associated with rapid fermentation and propionate formation [89]. While intensive feeding enhances fermentation efficiency and energy yield, extensive feeding sustains greater microbial diversity and a fiber-adapted microbiota more representative of the natural rumen ecosystem.

Dietary composition thus emerges as a primary determinant of ruminal structure and function. Pasture-based diets favour *Prevotella*, *Butyrivibrio*, and *Methanobrevibacter*, whereas high-concentrate feedlot diets promote *Ruminococcus* and *Fibrobacter*, both linked to cellulolytic activity [88]. Microbial succession also occurs throughout the bovine life cycle: neonatal calves exhibit high abundances of *Escherichia–Shigella* and *Corynebacterium*, while older animals show enrichment in LAB such as *Lactobacillus* and *Bifidobacterium*, taxa associated with improved feed efficiency and reduced methanogenesis [90].

Functional metagenomic analyses reveal robust expression of carbohydrate-active enzymes (CAZymes), including GH3, GH13, and GH43 families, which underpin polysaccharide degradation and efficient energy harvesting [86, 87, 91]. Synbiotic microbes further synthesize B-complex vitamins, contributing to host nutritional balance and immune modulation [92, 93].

Although microbial modulation has been consistently associated with improved feed efficiency and reduced methane emissions, the magnitude and direction of these effects vary depending on diet composition, animal genetics, and management practices. Understanding these multifactorial interactions is therefore essential for designing microbiota-targeted nutritional strategies that enhance ruminant productivity while mitigating environmental impacts.

### Biotechnological potential of probiotics in cattle production

Several LAB strains, including *E. faecium*, *L. plantarum*, *L. acidophilus*, *Limosilactobacillus mucosae*, and *B. subtilis*, have demonstrated promising effects across diverse cattle production systems. Supplementation with *E. faecium* 669 in pre-weaned calves increased average daily gain by up to 13.9% and improved feed intake without adverse effects [94, 95]. Similarly, long-term administration of *L. mucosae* CRL2069 in finishing cattle resulted in the highest daily weight gain (1.27 kg/day) and final body weight, highlighting its potential as a sustainable growth-promoting alternative [96].

Probiotic supplementation also modifies the gut microbial structure by enriching Clostridiaceae, Ruminococcaceae, and Bifidobacteriaceae, families associated with fiber degradation, volatile fatty acid (VFA) production, and intestinal homeostasis [97]. In mastitic cows, *L. plantarum* CM49 reduced *Streptococcus* and *Staphylococcus* abundance while restoring microbial diversity [98], suggesting that LAB can function as both gut modulators and immunobiotic agents with potential applications beyond nutrition.

From a food safety perspective, probiotics have shown preventive and sanitizing potential: *L. fermentum* CRL2085 supplementation reduced *E. coli* O157:H7 shedding [96], and *E. faecium* decreased *Eimeria* infection rates in young calves [94].

Immunomodulatory effects have been reported for synbiotic formulations combining LAB with yeast-derived prebiotics. These interventions improved ruminal epithelial integrity, elevated haptoglobin and leptin levels, and enhanced feed efficiency, carcass quality, and ruminal fermentation parameters [99]. Remarkably, their performance was comparable to that of conventional antimicrobials such as monensin and tylosin, underscoring their potential as viable antibiotic alternatives in ruminant nutrition [99].

Integrating prebiotics into synbiotic formulations is crucial to unlock the full potential of probiotics in cattle. The selection of appropriate prebiotic substrates determines colonization success and the persistence of beneficial taxa, making it a cornerstone of effective and sustainable feed additive design. Beyond biological benefits, there is growing commercial interest in these compounds, mirroring trends in swine and poultry sectors where prebiotic–probiotic blends are marketed as value-added products, such as Kormomix^®^ Rumin (LLC PO “Sibbiofarm”, Berdsk, Russia) [100] and Oreganol^®^ (Asteri Veterinary Medicines Industry Ltd., São Paulo, Brazil) [101].

Collectively, these findings emphasize that rationally designed synbiotic strategies, grounded in targeted prebiotic selection and robust LAB characterization, can simultaneously improve cattle health, productivity, and food safety, while supporting the development of innovative, antibiotic-free, and commercially viable feed solutions for sustainable ruminant production.

### Impact of prebiotic associations on cattle nutrition

Prebiotics derived from agro-industrial residues, inulin, yeast cell wall components, and macroalgae have shown significant impacts on ruminal microbiota composition, immune function, and animal productivity (Table 3). Fermented by-products such as rice straw [102] and palm kernel cake [103] increased bacterial diversity by enriching Bacteroidetes, Ruminococcaceae, and *Prevotella* while reducing Proteobacteria, indicating the establishment of a more stable and functionally balanced ruminal environment. Similarly, yeast cell wall fractions performed comparably to gentamicin in reducing *E. coli* and *Clostridium perfringens* populations, while simultaneously promoting *Lactobacillus* and *Bifidobacterium* proliferation [104].

**Table 3:** Effects of different prebiotics on digestibility, zootechnical performance, immune response modulation and intestinal microbiota in calves. Values are expressed as a percentage (%) increase or reduction compared to the control group, or as an absolute change, as described in the original studies

Prebiotics	Digestibility and metabolic improvements	Growth and production performance	Immune enhancement	Microbiota modulation	Studies
Beet pulp–rice straw silage (30%)	DMD + 4.1%; NDFD + 8.8%; ↑ SCFAs: Propionate + 15.3%; MUN − 7.1%; UN -5.5%; NH₃-N -4.1%	Milk yield + 4.9%; Milk protein + 17.4%	-	-	[105]
Fermented corn gluten meal	NH₃ -20%; MCP = 5.8%; ↑ SCFAs: Propionate + 24.6%; Diarrhea incidence − 30.8%	ADG + 8.8%; Concentrate intake + 8.3%	IgA + 11.4%; IFN-γ + 15.3%; IL-6 -9.1%;	↑ Bacteroidetes; ↓ Proteobacteria	[106]
Fermented palm kernel cake (3%)	CP + 0.6%; NDF − 25%; ADF − 56%	-	-	↑ α-diversity; ↑ Bacteroidetes, Fibrobacteres, Fibrobacteres, Spirochaetes, and Verrucomicrobia; ↑ *Prevotella*, *Treponema*, and *Succiniclasticum*	[103]
*S. boulardii* wall polysaccharides (500 mg/day)	-	BW + 3.95–4.90%; ADG + 28.49%; F/G -22.89%; Diarrhea − 18.54%	IgG + 51.97%; IL-10 + 45.45%; IL-1 -30.47%; IL-6 -28.17%; TNF-α -25.49%	↑ *Lactobacillus*, and *Bifidobacterium*; ↓ *E. coli*, *C. perfringens*, *Escherichia-Shigella*	[104]

*ADF* Acid Detergent Fiber, *ADG* Average Daily Gain, *BW* Body Weight, *CP* Crude Protein, *DMD* Dry Matter Digestibility, *F/G* Feed-to-gain Ratio, *MCP* Microbial Crude Protein, *MUN* Milk Urea Nitrogen, *NDF* Neutral Detergent Fiber, *NDFD* Neutral Detergent Fiber Digestibility, *TNF-α* Tumor Necrosis Factor Alpha, *UN* Urea Nitrogen

Beyond microbiota modulation, prebiotic supplementation confers notable immunological benefits. Increased levels of IgA, IgG, and IgM, accompanied by reductions in pro-inflammatory cytokines, have been observed following supplementation with fermented corn gluten meal and oregano essential oil [106, 107]. Early-life fiber administration further supports immune and metabolic programming by promoting colonization of *Prevotella*, *Shuttleworthia*, and *Selenomonas*, which enhance ruminal maturation and milk yield in adulthood [108].

Performance-oriented studies have reported higher average daily gain, increased milk protein content, and improved nitrogen utilization efficiency, alongside reduced feeding costs, when agro-industrial co-products such as brewer’s grains, yeast, beet pulp, and rice straw were incorporated into cattle diets [105, 109, 110]. While inulin enhances saccharolytic fermentation and SCFA production, fiber-rich residues act through gradual microbial adaptation, contributing to long-term ruminal stability and resilience.

Prebiotic-based interventions also yield environmental benefits, including reduced methane emissions [111] and the valorisation of agricultural residues within the feed chain. Their versatility enables tailored formulations that target growth performance, metabolic efficiency, or immune modulation across different production stages and management systems. Moreover, integrating prebiotics with probiotic strains in synbiotic formulations may further strengthen ruminal robustness and stability, amplifying the effects of microbiota-driven nutrition.

Collectively, these outcomes underscore the biotechnological relevance of prebiotic-based strategies within a circular livestock economy, where enhanced productivity, animal health, and environmental stewardship converge to define the next generation of sustainable ruminant nutrition systems.

## Nutrition biotechnology in fish production: impacts on intestinal health, immunity and productivity

In 2022, global aquaculture production reached a record 130.9 million tonnes, valued at US$312.8 billion, with finfish representing the largest share of total output. The term *finfish* refers specifically to true fish, excluding crustaceans and molluscs, with Nile tilapia ranking among the most widely farmed species worldwide [112]. China, India, Indonesia, Vietnam, and Norway led production, while other regions, including Africa and Oceania, continued to experience slower growth. In Latin America, countries such as Chile have consolidated highly competitive, export-oriented aquaculture industries, particularly in rainbow trout, achieving US$8.5 billion in aquatic animal exports in 2022, a 25% increase compared with the previous year [112].

Sustainable intensification strategies, centred on alternative feed ingredients, improved nutrient utilization, and circular resource flows, are driving the development of climate-smart aquaculture systems that enhance production efficiency while reducing environmental footprints. In parallel, global trade in aquatic animal products expanded to US$195 billion, with China, Norway, Vietnam, Ecuador, and Chile as the leading exporters, and the United States, China, Japan, the European Union, and the Republic of Korea serving as the primary import markets [112].

Within this evolving scenario, nutrition biotechnology has emerged as a transformative approach to optimize fish health, feed efficiency, and disease resistance. The integration of probiotics, prebiotics, and synbiotic formulations, often derived from sustainable bioresources, represents a cornerstone of modern aquaculture nutrition, aligning productivity goals with environmental responsibility and food safety in global fish farming systems.

### Microbiome profile and taxonomic overview in fish

Metagenomic studies have revealed the dynamic and host-specific nature of the fish gut microbiota, emphasizing its essential roles in digestion, immune regulation, and metabolic balance. A key genus across multiple species is *Cetobacterium*, which contributes to vitamin B12 biosynthesis and amino acid metabolism, thereby supporting host nutrition and intestinal stability [113–115].

In Nile tilapia (*Oreochromis niloticus*) raised under standard aquaculture conditions, the gut microbiota is composed of Proteobacteria, Fusobacteria (mainly *Cetobacterium*), Firmicutes, Bacteroidetes, and Actinobacteria [116, 117]. Similarly, in rainbow trout (*Oncorhynchus mykiss*), Proteobacteria and Firmicutes dominate, with additional representation from Actinobacteria and Bacteroidetes. The most frequently detected genera, *Aeromonas*, *Pseudomonas*, and *Clostridium*-related taxa, are linked to nutrient absorption, immune modulation, and intestinal resilience [118, 119].

Environmental and dietary factors markedly influence microbial composition. In tilapia, exposure to salinity stress increased the abundance of *Cetobacterium somerae*, *Bacillus*, *Halomonas*, and *Vibrio*, indicating adaptive responses and potential endogenous probiotic functions [114]. In rainbow trout, diet-induced and temperature-related shifts were observed: Mycoplasmataceae dominated under lower temperatures, whereas *Enterobacteriaceae* proliferated under warmer, plant-based diets [120]. Nutritional interventions, such as supplementation with *P. acidilactici* and galactooligosaccharides (GOS), reduced *Mycoplasma* abundance while enriching *Pediococcus*, *Massilia*, and *Weissella*, taxa associated with gut integrity, lipid metabolism, and immune stimulation [121].

In carp (*Cyprinus carpio*) and zebrafish (*Danio rerio*), *Cetobacterium* and *Aeromonas* persist as core synbionts even under recirculating aquaculture systems, while ammonia-oxidizing *Nitrosomonas* contributes to nitrogen processing within gill-associated microbiota [122]. Moreover, several marine LAB isolates, including *Lactococcus lactis*, *Enterococcus mundtii*, and *Latilactobacillus sakei*, have demonstrated antimicrobial activity and biosafety for probiotic use [123], reinforcing the potential of LAB as biocontrol and microbiota-modulating agents in aquaculture.

Collectively, these findings underscore the ecological and biotechnological importance of the fish gut microbiome as a driver of nutritional efficiency, immune competence, and disease resistance. Understanding these taxonomic and functional dynamics provides the foundation for microbiome-informed feed design and precision aquaculture, advancing both animal performance and environmental sustainability.

### Biotechnological potential of probiotics in fish production

Probiotics have demonstrated significant benefits in aquaculture, particularly in Nile tilapia, where they improve growth performance, feed efficiency, and intestinal morphology [124]. In common carp exposed to heavy metals, *Lactococcus lactis* provided detoxifying and immunoprotective effects by removing contaminants and preserving tissue integrity, even in the absence of stable intestinal colonization [125]. These results emphasize the multifaceted actions of probiotics, which extend beyond microbial competition to include antioxidant, detoxification, and immunomodulatory mechanisms.

Yeast-derived prebiotics, such as *Saccharomyces cerevisiae* and *Cyberlindnera jandinii*, have also been shown to reshape the intestinal microbiota of carp by promoting *Cetobacterium* dominance and reducing opportunistic pathogens like *Aeromonas* and *Citrobacter*, in some cases outperforming probiotics alone [126]. Similarly, supplementation with FOS enhanced immune responses in *Labeo fimbriatus* when combined with *B. subtilis*, increasing phagocytic activity, lysozyme levels, and microvillus density, improving survival rates following *Aeromonas hydrophila* challenge [127].

Collectively, FOS and yeast derivatives, whether administered individually or synergistically with probiotics, represent sustainable biotechnological strategies to enhance growth, immune competence, and disease resistance in fish. Such combinations align with the global trend toward eco-efficient and antibiotic-free aquaculture, reinforcing the role of microbiota-targeted nutrition in modern production systems.

The application of commercial probiotics and synbiotic has further validated these benefits across multiple finfish species. Examples include the synbiotic Bio-Aqua^®^ (*Zist Darman Mahan Company*, Tehran, Iran) in Caspian trout [128]; the probiotic products AquaStar^®^ (*DSM-Firmenich*, Basel, Switzerland) and EM^®^ (*EM Research Organization*, Okinawa, Japan) in Nile tilapia [129]; and experimental synbiotic formulations for rainbow trout under development [130]. These findings highlight the expanding commercial and technological relevance of synbiotic in aquaculture, offering viable, evidence-based alternatives to antibiotics while supporting gut health, pathogen resistance, and optimal growth performance.

### Impact of prebiotic associations on fish nutrition

The use of prebiotics in aquaculture has gained increasing attention as a multifaceted biotechnological strategy to support intestinal health, enhance growth performance, modulate immune function, and mitigate environmental impacts (Table 4). Although these benefits have been consistently reported across a wide range of fish species, the magnitude and nature of the responses depend strongly on species-specific physiology, feeding behaviour, and production system characteristics. This variability underscores the importance of tailoring prebiotic applications to the biological and ecological context of each aquaculture model.

**Table 4:** Effects of different prebiotics on digestibility, zootechnical performance, immune response modulation, intestinal microbiota, and morphology in tilapia and rainbow trout. Values are expressed as a percentage (%) increase or reduction compared to the control group, or as an absolute change, as described in the original studies

Fish groups	Prebiotics	Digestibility and metabolic improvements	Growth and production performance	Immune enhancement	Microbiota modulation	Morphology	Studies
Tilapia	FOS (1–4 g/kg)	-	BW + 9.8%	ALP (50.7 to 65.7 U/L); ACP (111.69 to ~ 125 U/L); LZM (421.9 to 451.7 U/L)	↑ LAB and *Bacillus*; no change in *E. coli*	VH + 47; VW 85.33 to 54.67 μm	[131]
	Inulin (5 g/kg) or Jerusalem artichoke (5–10 g/kg)	-	BW + 10–14%; FE + 9–15%	-	↑ LAB, *Bifidobacterium*; ↓ *Vibrio*, and yeast/fungi	VH + 91 μm; Goblet cells + 38%	[132]
	MOS (5 g/kg, high-carb diet)	Blood glucose − 25%; hepatic glycogen − 27%; ↑ insulin GLP-1	Body length + 7%; BW + 19%	↑ TGFβ; ↓ NF-κB, IL1β, IL6, IL8	↑ Actinobacteria; Decreases Proteobacteria and Fusobacteria	-	[133]
Rainbow trout	Cello-oligosaccharides (0.5–1.5%, birch-derived	-	BW ~ 132–144% (NS); SGR ~ 1.77–1.89 (NS); Survival 100%	↑ c3 and trend for c-type lectin and TLR2	↑ Ruminococcaceae, Bacillaceae, and Lactobacillaceae	-	[134]
	*Hermetia illucens* meal and poultry by-product meal	-	BW + 11.8%; SGR + 7.6%; FCR − 8.7%	↑ TLR1; ↓ of IL1b, TNF-α, NF-κB, MYD88	-	VH + 65%; Reduced enteritis with ≥ 30% HM or PBM	[135]
	XOS (5–10 g/kg, plant-derived)	↑ Lipase (119 to 417 U/mgprot), and amylase (2.53 to 3.61 U/mgprot)	BW 50.3 to 54.9 g; WGR 144.4% to 162.2%; SGR 1.60 to 1.72; FCR 1.16 to 0.08 (NS)	↑ Claudin-1 and ZO-1; ↓ TNF-α, IL-6, and IL-10	↑ *Lactobacillus*; ↓ *E. coli*	VH + 53%; Muscle thickness 57 to 95 μm CD 92 to 187 μm	[136]

*ACP* Acid Phosphatase, *ADC-CP* Apparent Digestibility Coefficient of Crude Protein, *ALP* Alkaline Phosphatase, *BW* Body Weight, *CAT* Catalase, *FCR* Feed Conversion Ratio, *FE* Feed Efficiency, *GLP-1* Glucagon-Like Peptide-1, *GPX* Glutathione Peroxidase, *LR* Lipid Retention, *LZM* Lysozyme, *MDA* Malondialdehyde, *MYD88* Myeloid Differentiation Primary Response 88, *NBT* Nitroblue Tetrazolium, *PR* Protein Retention, *SGR* Specific Growth Rate, *SOD* Superoxide Dismutase, *TLR* Toll-Like Receptor, *TNF-α* Tumor Necrosis Factor alpha, *VH* Villus Height, *VH/CD* Villus Height to Crypt Depth Ratio, *VW* Villus Width, *WBC* White Blood Cells, *WGR* Weight Gain Rate, *ZO-1* Zonula Occludens-1

#### Tilapia (*Oreochromis niloticus* and hybrids)

Nile tilapia has been extensively investigated regarding prebiotic and synbiotic supplementation, with consistent evidence of morphological, metabolic, and physiological benefits. Diets enriched with inulin or Jerusalem artichoke increased villus height and goblet cell density throughout the intestine, enhancing absorptive capacity and mucosal protection [132].

Similarly, MOS alleviated diet-induced hepatic steatosis by downregulating *srebp*1 and *fas* expression and reducing triglyceride accumulation [133]. These findings are particularly relevant to intensive tilapia farming, where high-energy diets predispose fish to lipid deposition and compromised liver function [137].

Beyond metabolic regulation, behavioural and welfare indicators also show improvement following prebiotic supplementation. Administration of β-glucans conferred complete survival under hypoxic challenge and lowered blood glucose levels, indicating enhanced stress resilience and energy conservation under intensive rearing conditions [138].

Synbiotic formulations combining *B. subtilis* with β-glucans further increased resistance against *A. hydrophila* and reduced mortality, supporting their use as functional antibiotic alternatives [139]. Moreover, the inclusion of *Aspergillus oryzae* together with β-glucans decreased malondialdehyde levels by approximately 35%, while enhancing antioxidant enzyme activity, demonstrating their capacity to counteract oxidative stress and sustain cellular homeostasis [140].

Collectively, these studies establish prebiotic and synbiotic supplementation as effective tools to enhance gut structure, metabolic stability, and disease resistance in tilapia, providing a foundation for more resilient, health-oriented aquaculture systems.

#### Rainbow trout

Rainbow trout consistently exhibit positive responses to prebiotic supplementation. Xylo- and cello-oligosaccharides enhance Toll-like receptor expression [134] and increase villus height and intestinal muscularis thickness, reinforcing gut barrier integrity [136]. These structural adaptations are accompanied by microbiota shifts toward beneficial taxa, including *Ruminococcaceae*, *Lactobacillus*, and *Bifidobacterium*, while opportunistic pathogens such as *Aeromonas* and *Vibrio* are suppressed [134].

Dietary inulin further improves microvilli architecture and induces hepatic transcriptomic remodelling, upregulating genes involved in fatty acid metabolism and receptor-mediated signalling, whereas MOS supplementation elicits tissue-specific metabolic effects in the liver and muscle [141]. Combined dietary formulations integrating FOS, probiotics, and yeast extracts have been shown to enhance growth performance, digestive enzyme activity, and immune parameters (e.g., lysozyme, complement, β-defensin), while increasing resistance against pathogens such as *A. hydrophila* and *Yersinia ruckeri* [142].

Supplementation with inulin at 2% promotes protein deposition, mucosal immunity (lysozyme, IgM, protease), and survival in challenged fry [143]. Similarly, synbiotic formulations combining *Lactobacillus delbrueckii* with *Asparagus officinalis* root extracts optimize feed utilization, antioxidant responses, and resilience under crowding stress [144].

These effects mirror those observed in *Nile tilapia*, where prebiotic and synbiotic interventions likewise enhance intestinal morphology, immune function, and stress tolerance. Collectively, these findings highlight the cross-species potential of microbiota-targeted nutritional strategies to improve health and performance under intensive aquaculture conditions, advancing sustainable and antibiotic-free fish production.

#### Other finfish species

Significant physiological, immunological, and metabolic responses to prebiotic supplementation have been reported across a wide range of aquaculture species, demonstrating consistent improvements in growth performance, gut health, immune function, and resilience, while underscoring the potential of tailored nutritional strategies in fish farming.

In freshwater species, prebiotic supplementation has consistently enhanced growth, gut microbiota diversity, and immune parameters. For example, in Reba carp (*Cirrhinus reba*), the inclusion of brewers’ spent grain improved growth performance and elevated serum enzyme activity without inducing hepatic histopathology. Microbiome analysis revealed increased diversity and higher abundances of *Bacillus* and LAB [145]. Similarly, in Ña-fish (*Schizothorax prenanti*), konjac oligosaccharides stimulated innate immunity by increasing lysozyme and acid phosphatase activity and reducing *Aeromonas* loads [146]. Complementary gene expression analyses in this and other species, such as hybrid sturgeon (*Acipenser baeri vs. A. schrenckii*), revealed upregulation of immune-related genes including *C3*, lysozyme, and pro-inflammatory cytokines, indicating robust immune priming [146, 147].

Warm-water species, such as striped catfish (*Pangasianodon hypophthalmus*), responded favourably to GOS and β-glucan supplementation, exhibiting increased final body weight and improved feed conversion efficiency [148]. In carnivorous freshwater species such as largemouth bass (*Micropterus salmoides*), resistant starch improved growth performance, reduced hepatic lipid accumulation, promoted lipid catabolism, enhanced SCFA production, and suppressed pathogenic bacteria, including *E. coli* [149].

Prebiotic effects also extend to marine finfish, though responses often show greater sensitivity to inclusion levels. In turbot (*Scophthalmus maximus*), xylan supplementation produced dose-dependent effects: 1.25% inclusion enhanced intestinal integrity by upregulating *TGF-β* and tight-junction proteins while suppressing IL-1β, whereas 5% inclusion disrupted microbial balance, underscoring the importance of precise dose optimization [150]. A synergistic response was observed in croaker (*Nibea coibor*), where combined GOS and sorbitol supplementation significantly increased weight gain and feed efficiency [151]. Likewise, in gilthead sea bream (*Sparus aurata*), MOS improved intestinal morphology, particularly microvilli architecture, resulting in enhanced nutrient absorption [152].

Overall, prebiotic supplementation benefits both intensive and semi-intensive aquaculture systems, across freshwater and marine species, by enhancing gut health, immunity, and metabolic stability. Intensive systems exhibit stronger responses due to higher stocking densities and nutrient loads, whereas semi-intensive systems gain through improved feed efficiency and disease resistance. Moreover, sourcing prebiotics from agro-industrial by-products exemplifies circular economy principles, transforming agricultural waste into functional, value-added feed inputs for sustainable aquaculture.

## Nutrition biotechnology in shrimp production: impacts on intestinal health, immunity and productivity

The intensive farming of Whiteleg shrimp (*Penaeus vannamei*) and giant tiger prawn (*Penaeus monodon*), now widely adopted across global aquaculture, faces persistent challenges associated with high stocking densities, which favour bacterial and viral disease outbreaks and compromise production stability [153]. Whiteleg shrimp remains the dominant farmed shrimp species, reaching 6.8 million tonnes in 2022. Global demand for shrimp products remains strong, particularly in China and the European Union, while consumption in the United States showed a slight decline [112].

In the same year, global exports of shrimp and prawns reached USD 32.3 billion, representing an 8.7% increase over 2021, driven primarily by expanding aquaculture output. Ecuador emerged as the world’s largest shrimp exporter, surpassing India and Vietnam, with USD 10.1 billion in exports, while Vietnam exported USD 11.2 billion in aquatic products, farmed shrimp [112]. China, a leading producer and exporter, accounted for 12% of global aquatic animal exports, reflecting the strategic role of processed shrimp and other farmed species in international trade [112].

Within this rapidly evolving production landscape, nutrition biotechnology has become a central pillar of disease prevention and performance optimization in shrimp aquaculture. The integration of probiotics, prebiotics, and synbiotic has demonstrated strong potential to improve gut health, enhance immune responsiveness, and increase zootechnical efficiency, while reducing reliance on antibiotics and supporting the transition toward resilient, health-oriented, and environmentally responsible shrimp production systems.

### Microbiome profile and taxonomic overview in shrimp

Metagenomic studies have shown that the shrimp gut microbiota, dominated by Proteobacteria, Firmicutes, Actinobacteria, and Bacteroidetes, plays pivotal roles in nutrient metabolism, immune regulation, and overall productivity [154, 155]. Beneficial genera such as *Lactobacillus*, *Bacillus*, *Ruegeria*, and *Ilumatobacter* are commonly enriched in intensive farming systems, where they support SCFA production, vitamin biosynthesis (notably B12 and K2), and amino acid metabolism [155].

Interestingly, fast-growing shrimp often exhibit lower microbial diversity but higher abundances of SCFA-producing taxa such as *Coprococcus comes*, *Oscillibacter*, and *Clostridium phoceensis* [156], suggesting that growth efficiency may be associated with functional specialization rather than taxonomic diversity. Conversely, dysbiosis, typically characterized by excessive *Vibrio* proliferation, has been linked to reduced digestive efficiency and greater disease susceptibility.

Probiotic interventions have shown clear restorative effects on gut balance. Strains such as *Bacillus pumilus*, *Ruegeria atlantica*, and *Celeribacter indicus* successfully reestablish microbial homeostasis, suppress Vibrio spp., and reduce mortality under pathogen challenge [154]. Likewise, LAB including *L. plantarum* and *P. pentosaceus* have been associated with enhanced gut stability, improved digestive performance, and accelerated growth.

Collectively, these findings highlight the functional centrality of the shrimp gut microbiome as both a determinant of metabolic efficiency and a target for biotechnological intervention. Understanding the compositional and functional dynamics of these communities provides the foundation for precision microbiome management in shrimp aquaculture, enabling strategies that combine probiotic, prebiotic, and synbiotic formulations to promote resilient and disease-resistant production systems.

### Biotechnological potential of probiotics in shrimp production

Probiotic supplementation has demonstrated substantial improvements in growth performance, feed efficiency, immune competence, and pathogen resistance in shrimp aquaculture. In Whiteleg shrimp, *P. pentosaceus* MR001 increased weight gain, improved the feed conversion ratio, and upregulated innate immune genes including *prophenoloxidase* (*proPO*), *Toll-like receptor* (*LvToll*), and *transglutaminase* (*TGase*), resulting in a 40% higher survival rate following *Vibrio parahaemolyticus* challenge [157]. Similarly, *L. plantarum* L20 provided 80% survival in shrimp exposed to Acute Hepatopancreatic Necrosis Disease (AHPND), demonstrating its efficacy as a prophylactic immunobiotic [158].

A multi-strain probiotic consortium comprising *Ruegeria lacuscaerulensis*, *Nioella nitratireducens*, *B. subtilis*, and *Streptomyces euryhalinus* effectively delayed mortality and reduced cumulative death rates from 30.4% to 5.2% under pathogen pressure [159]. Similarly, synbiotic formulations combining *L. acidophilus* with *Moringa oleifera* extracts improved growth, feed efficiency, and immune parameters, while reducing pathogen loads and oxidative stress markers [160].

The commercial application of probiotic and prebiotic products is now expanding beyond finfish to shrimp aquaculture. Examples include the prebiotic Sanacore^®^ GM (Adisseo, Dendermonde, Belgium) [161] and the probiotic Aqua Fortuna Probiotic (AFPB) (Aqua Fortuna Biotech Co., Ltd., Keelung City, Taiwan) [162], both of which have demonstrated beneficial effects on growth performance, immune modulation, and disease resistance.

Collectively, these findings highlight the biotechnological versatility of probiotic and synbiotic formulations in shrimp production. By reinforcing intestinal integrity, modulating host-microbiota-pathogen interactions, and reducing dependence on antibiotics, these interventions represent a cornerstone of sustainable, health-oriented shrimp aquaculture.

### Impact of prebiotic associations on shrimp nutrition

Prebiotics such as β-glucans, FOS, and agro-industrial residues (e.g., *Agave lechuguilla* bagasse, *Eleutherine bulbosa*) have consistently improved gut health, immune regulation, and growth performance in shrimp (Table 5). Supplementation with inulin at 5–20 g/kg enhanced growth, survival, and resistance to both *Vibrio parahaemolyticus* and white spot syndrome virus, while increasing gut microbial diversity and SCFA production [163]. These outcomes are mediated through enrichment of SCFA-producing taxa such as *Lactobacillus*, *Bacillus*, and *Ruegeria*, which collectively enhance nutrient metabolism, epithelial integrity, and immune responsiveness [154, 155].

**Table 5:** Effects of different prebiotics on digestibility, zootechnical performance, immune response modulation, intestinal microbiota, and morphology in shrimp. Values are expressed as a percentage (%) increase or reduction compared to the control group, or as an absolute change, as described in the original studies

Prebiotics	Digestibility and metabolic improvements	Growth and production performance	Immune enhancement	Microbiota modulation	Morphology	Studies
β-glucan (0.1–0.4%)	↑ Amylase, lipase, protease, and KEGG pathways for starch/sucrose, galactose, and carbohydrate digestion/absorption	BW + 33%; FBW 2.36 vs. 1.91 g; SGR 6.42 vs. 5.68%/d	↑ ACP, ALP, and expression of antimicrobial peptides	↑ *Lactobacillus*; ↓ *Vibrio*, *Rheinheimera*, and *Demequina*	-	[164]
*Eleutherine bulbosa* powder (12.5 g/kg)	ProtD + 10%; DT + 5.8%	FW + 24%; WG + 50%; SGR + 39%; FCR − 21%	-	↑ Diversity (Shannon + 13%) and OTUs (+ 45%)	Microvilli density + 138% Perimeter ratio + 59%	[165]
Flavonoid-enriched extract of Agave lechuguilla bagasse (0.1–0.6%)	-	0% Mortality in 0.3–0.6% groups after Vibrio parahaemolyticus challenge (72 h)	-	Shift from Alphaproteobacteria to Gammaproteobacteria, Bacteroidetes, and Verrucomicrobia; ↑ Rhodobacteraceae; ↓ Vibrionaceae	-	[166]
FOS (2–4 g/kg)	↑ Amylase and lipase activity	FW + 17.3%; WGR + 32.1%; FCR − 27%; Survival + 10%	↑ SOD, ALP, and ACP; Decreases MDA	-	-	[167]
FOS (water administration)	↑ Protease activity	BW + 40%; Length + 18%; Survival + 15%	-	↓ *Vibrio* spp. and *Pseudomonas* spp.	-	[168]
Yeast Cell Wall (0.5–1.0%)	-	Survival + 23%; BW + 4.2%; SGR + 1.6%; FCR − 48%; Productivity + 67%	-	OTUs (1.0% YCW) + 9.2% vs. 1.9% (control); *Bacillus* (0.43% for 7.7%); *Sphingobium* (0.37% for 19.4%); no dysbiosis	-	[169]

*ACP* Acid Phosphatase, *ALP* Alkaline Phosphatase, *BW* Body Weight, *DMD* Dry Matter Digestibility, *FBW* Final Body Weight, *FCR* Feed Conversion Ratio, *MDA* Malondialdehyde, *OTUs* Operational Taxonomic Units, *ProtD* Protein Digestibility, *SGR* Specific Growth Rate, *SOD* Superoxide Dismutase, *SR* Survival Rate, *WG* Weight Gain, *WGR* Weight Gain Rate

β-glucans further contribute broad-spectrum immunostimulatory effects, including increased phenoloxidase activity, respiratory burst, lysozyme secretion, antimicrobial peptide synthesis, and total haemocyte counts [168]. Similarly, dietary inclusion of mannan-oligosaccharides (MOS; 0.5% w/w) favourably reshaped the intestinal microbiota by increasing Actinobacteria and reducing Proteobacteria and other potential pathogens, while concurrently improving survival rates by 30% in intensive production systems [170].

These results collectively illustrate how prebiotic supplementation complements the natural gut microbiome, reinforcing microbial equilibrium, nutrient assimilation, and host resilience against disease. However, the mechanisms of action appear to vary according to substrate type, host condition, and farming intensity, emphasizing the need for mechanistic validation and cost-effectiveness assessments to guide industrial implementation.

Integrating the microbiome’s functional landscape with observed physiological outcomes provides a conceptual framework for rationally designed prebiotic formulations in shrimp aquaculture, bridging microbial ecology, host physiology, and sustainable production.

## The black soldier fly (*Hermetia illucens*) as a novel source of bioactives for animal nutrition

Recent advances in animal nutrition biotechnology have expanded the definition of *prebiotics* beyond traditional plant-derived substrates such as inulin and FOS. Within this broader context, the black soldier fly (BSF), particularly its larval stage (*Hermetia illucens* larvae, BSFL), has emerged as a multifunctional bioactive resource. Initially investigated as a sustainable protein alternative, BSFL and their derivatives (e.g., defatted meals, prepupae) have demonstrated prebiotic-like activities owing to their content of chitin [171], SCFAs [172], and antimicrobial peptides [173].

In poultry, BSF inclusion modulates the gut microbiota while maintaining zootechnical performance. Diets containing 3–5% BSFL preserved feed efficiency and increased beneficial taxa such as *Lactobacillus*, while reducing *Salmonella* in the caeca of broilers [174]. Higher inclusion levels (up to 20%) enriched *Roseburia*, a butyrate-producing genus linked to intestinal health and immune modulation [175]. In laying hens, BSFL promoted microbial diversity, enhancing the abundance of *Lactobacillus*, *Bacteroides*, and *Enterococcus*, taxa associated with nutrient absorption and gut resilience [176]. The chitin fraction in BSFL also supports the proliferation of *Flavonifractor plautii* and *Christensenella minuta*, both SCFA producers that reinforce intestinal barrier integrity [172].

In aquaculture, BSF-derived ingredients have gained traction as functional feed additives. In *Dicentrarchus labrax* (European seabass), BSF meal enhanced microbial richness, favouring *Lactobacillus* and *Bacillus*, while preserving gut morphology and growth [177]. Similarly, in *Sparus aurata* (gilthead sea bream), BSFL inclusion stimulated chitin-degrading microbiota and promoted hepatic health [178]. In Nile tilapia, enzyme-assisted digestion with chitinase improved microbial balance by increasing *Firmicutes* and reducing *Proteobacteria* [179]. In rainbow trout, inclusion levels up to 30% enhanced gut structure and reduced inflammatory markers [135], whereas sturgeon displayed reduced palatability at similar levels, highlighting species-specific responses [180].

While the beneficial outcomes of BSF-derived prebiotics and probiotics have been consistently demonstrated in several fish species, variability among taxa underscores the importance of species-tailored nutritional strategies that account for dietary physiology, environmental context, and farming intensity. Such targeted approaches are essential to fully optimize gut health, growth performance, and production sustainability across diverse aquaculture systems.

Overall, BSF represents a convergence point between nutrition, gut health, and circular bioeconomy. Its prebiotic-like effects consistently promote beneficial microbial taxa across animal groups, enhancing intestinal resilience, metabolic efficiency, and the functional value of the resulting biomass. While highly promising, broader industrial adoption requires validation under commercial-scale conditions, encompassing processing costs, regulatory frameworks, and comparative efficacy relative to conventional protein and prebiotic sources.

**Fig. 2:**
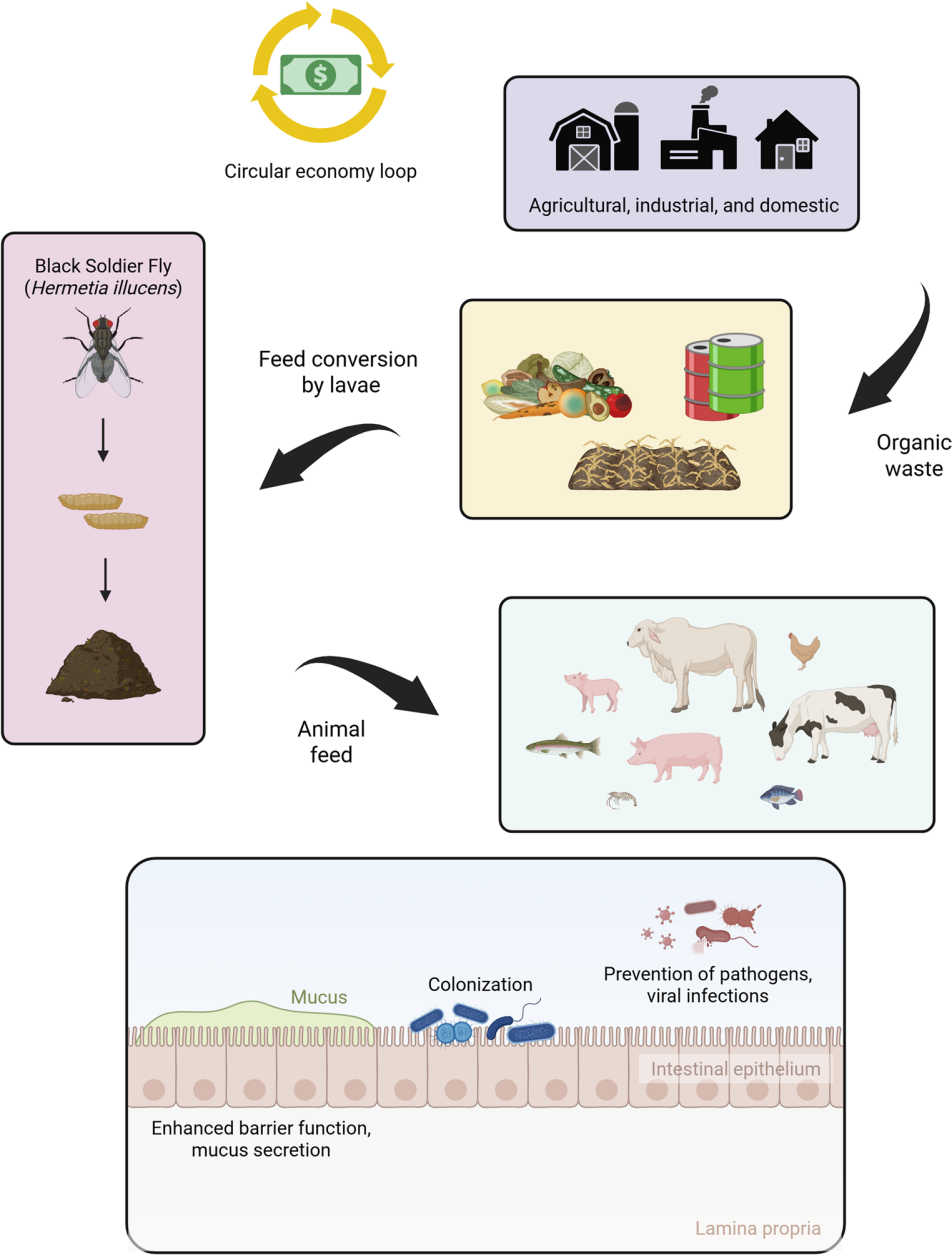
Black Soldier Fly (*Hermetia illucens*) larva meal as an emerging prebiotic and alternative protein. Converts organic waste into high-value nutrients. Promotes gut health, beneficial microbiota, and immune modulation in poultry and fish. A circular economy approach

Beyond zootechnical performance, BSF-based formulations contribute directly to the United Nations SDGs, particularly SDG 2 (Zero Hunger) and SDG 12 (Responsible Consumption and Production) [181], by improving nutrient utilization, reducing reliance on fishmeal and soybean-based feeds, and valorising organic waste streams [182, 183] (Fig. 2). These integrated benefits position BSF as a next-generation bioactive platform bridging sustainable feed innovation and microbiome-driven animal health.

## Synthesis, future perspectives, and research directions

**Fig. 3:**
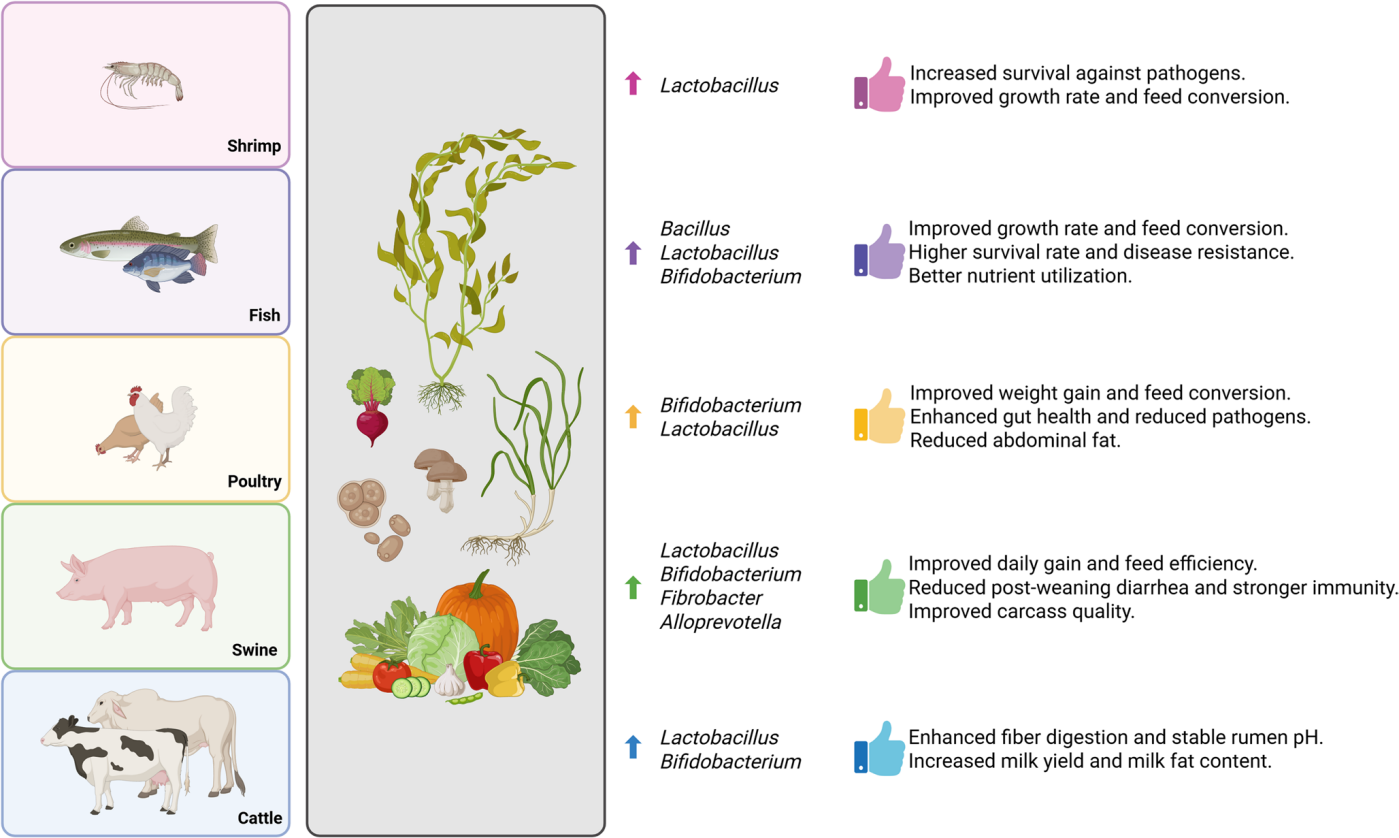
Representative overview of prebiotic effects on beneficial bacterial genera and performance outcomes in different animal species. The figure illustrates species-specific responses, highlighting the stimulation of key gut microbes (e.g., *Lactobacillus*, *Bifidobacterium*) and their association with improved growth, feed efficiency, nutrient utilization, and resistance to pathogens across aquaculture, poultry, swine, and ruminant systems

The growing body of evidence underscores the pivotal role of prebiotics and probiotics in shaping intestinal health, immune regulation, and productive performance across diverse animal production systems exposed to different dietary components and functional feed ingredients (Fig. 3; Tables 1, 2, 3, 4 and 5). Their capacity to modulate the gut microbiota underpins improvements in feed efficiency, growth performance, and disease resistance, while reducing antibiotic dependency and environmental footprint [184].

From a sustainability perspective, the inclusion of fermentable fibers, insect larvae, and agro-industrial co-products aligns livestock production with the principles of the circular economy and several United Nations SDGs, particularly those related to responsible consumption (SDG 12), climate action (SDG 13), and food security (SDG 2) [185–187].

### Research priorities

Future research should focus on three interrelated fronts:

1. Precision nutrition and meta-omics integration: High-resolution *meta-omics* approaches (metagenomics, metatranscriptomics, metabolomics) will be essential to elucidate diet–microbiome–host interactions, enabling the design of targeted prebiotic–probiotic formulations tailored to specific species, production systems, and life stages [188].
2. Large-scale validation under commercial conditions: Longitudinal trials incorporating genetic, environmental, and management variability are required to confirm laboratory findings at farm scale, bridging the gap between experimental research and field applicability [189].
3. Life-cycle and techno-economic assessment: Integrating environmental impact models and cost-benefit analyses will be crucial to evaluate economic viability, carbon balance, and regulatory feasibility of biofunctional feed additives [186].

### Market outlook

The global probiotics market was valued at US$ 74.3 billion in 2023 and is projected to grow at a compound annual growth rate (CAGR) of 14.1% through 2030 [190]. Similarly, the animal feed probiotics sector reached US$ 5.0 billion in 2024, expanding at a CAGR of 5.1% [191]. The prebiotics market is following an even steeper trajectory, estimated at US$ 8.5 billion in 2023 and projected to reach US$ 21.8 billion by 2030 (CAGR 14.3%) [192]. In contrast, the animal prebiotics intestinal health market remains smaller, valued at US$ 593.7 million in 2024, but with a promising CAGR of 9.6% through 2030 [193].

This imbalance between human- and animal-targeted formulations highlights a major economic and innovation gap, revealing opportunities for biotechnological expansion and market diversification in animal nutrition. The rising demand for functional feed ingredients and microbiome-based modulation in livestock and aquaculture provides fertile ground for industrial development.

### Path forward

As precision nutrition, meta-omics insights, and commercial validation deepen our understanding of species-specific gut ecosystems, there is increasing scope to develop customized biofunctional feeds for poultry [31, 32], pigs [63, 64], cattle [92, 93], fish [120–122], and shrimp [161, 162]. These developments will drive biotechnological innovation, value-added feed production, and market expansion across animal production sectors.

Advancing these research priorities will accelerate the transition toward microbiome-informed, antibiotic-free, and climate-resilient livestock and aquaculture systems. By leveraging fermentable fibers, agro-industrial by-products, and novel functional ingredients such as the black soldier fly, future animal nutrition will integrate health, efficiency, and environmental stewardship, bridging the gap between animal well-being and planetary sustainability.

## Conclusion

The integration of prebiotics and probiotics into animal nutrition represents a transformative biotechnological strategy to enhance gut health, immune function, and productivity across diverse production systems. Evidence from poultry, swine, cattle, fish, shrimp, and emerging insect-based models such as the black soldier fly demonstrates the ability of these biofunctional additives to modulate the gut microbiota, strengthen host resilience, and reduce antimicrobial dependency, while simultaneously valorising agro-industrial by-products and advancing circular economy principles.

Despite substantial progress, important knowledge gaps remain. Future research should prioritize the standardization of experimental designs, the application of integrated meta-omics approaches to decipher diet-microbiome-host interactions, and the validation of findings under commercial conditions through large-scale, longitudinal trials. Furthermore, life cycle assessments and techno-economic analyses are crucial to ensure that these innovations are not only biologically effective but also economically viable and environmentally sustainable.

Collectively, these advances will drive the transition toward antibiotic-free, climate-resilient, and resource-efficient animal production systems. By integrating microbiota-targeted nutrition, precision feeding, and functional feed additives, producers can enhance animal health, growth, and welfare while mitigating environmental impacts such as greenhouse gas emissions and nutrient losses. Such strategies directly align livestock production with global sustainability frameworks and consumer demand for ethical and safe food systems.

In summary, microbiota-driven nutrition based on prebiotics and probiotics offers a scalable pathway toward sustainable and welfare-oriented livestock and aquaculture. By improving intestinal integrity, immune competence, and nutrient utilization, these interventions enhance productivity and disease resilience while embedding circularity and waste valorisation, for instance, through black soldier fly derivatives and agro-industrial residues, into feed innovation. Bridging microbiome science with food safety, environmental stewardship, and One Health principles will be essential to secure the long-term resilience of animal production systems. Ultimately, microbiota-based functional nutrition provides a scientifically grounded and economically feasible route toward ethical, climate-responsible, and safe food production for a growing global population.

## Abbreviations

ACP: Acid Phosphatase
ADC-CP: Apparent Digestibility Coefficient of Crude Protein
ADF: Acid Detergent Fiber
ADF I: Acid Detergent Fiber Intake
ADG: Average Daily Gain
AHPND: Acute Hepatopancreatic Necrosis Disease
ALP: Alkaline Phosphatase
AP: Apple Pomace
BSF: Black Soldier Fly
BSFL: Black Soldier Fly Larvae
BW: Body Weight
CAGR: Compound Annual Growth Rate
CAT: Catalase
CAZymes: Carbohydrate-Active Enzymes
CD: Crypt Depth
DMD: Dry Matter Digestibility
FAO: Food and Agriculture Organization
FBW: Final Body Weight
FCR: Feed Conversion Ratio
FE: Feed Efficiency
F/G: Feed-to-gain Ratio
FOS: Fructooligosaccharide
GE: Gross Energy
GLO: Globulin
GLP-1: Glucagon-Like Peptide-1
GOS: Galactooligosaccharides
GPX: Glutathione Peroxidase
GSH: Reduced Glutathione
H:L: Heterophil/Lymphocyte ratio
IBD: Antibodies Against Infectious Bursal Disease
IgA: Immunoglobulin A
IgG: Immunoglobulin G
IgM: Immunoglobulin M
IL-10: Interleukin-10
IL-1β: Interleukin-1β
IL-4: Interleukin-4
IFN-γ: Interferon-γ
ISAPP: International Scientific Association for Probiotics and Prebiotics
LAB: Lactic Acid Bacteria
LDL-C: Low-Density Lipoprotein Cholesterol
LR: Lipid Retention
LWG: Live Weight Gain
LZM: Lysozyme
MCP: Microbial crude protein
MDA: Malondialdehyde
MOS: Mannan-Oligosaccharides
MUN: Milk urea nitrogen
MYD88: Myeloid Differentiation Primary Response 88
NBT: Nitroblue Tetrazolium
NDFD: Neutral detergent fiber digestibility
NDF: Neutral Detergent Fiber
NDV: Antibodies Against Newcastle Disease Virus
OTUs: Operational Taxonomic Units
PB: Crude Protein
ProtD: Protein Digestibility
PR: Protein Retention
SBP: Sugar Beet Pulp
SCFA: Short-Chain Fatty Acid
SDGs: Sustainable Development Goals
SGR: Specific Growth Rate
SH: Soy Hulls
SOD: Superoxide Dismutase
SR: Survival Rate
TAC: Total Antioxidant Capacity
TG: Triglycerides
TLR: Toll-Like Receptor
TNF-α: Tumor Necrosis Factor alpha
TP: Total Protein
UN: Urea nitrogen
VFA: Volatile Fatty Acid
VH/CD: Villus Height / Crypt Depth Ratio
VH: Villus Height
VW: Villus Width
WBC: White Blood Cells
WGR: Weight Gain Rate
WG: Weight Gain
XOS: Xylooligosaccharides
ZO-1: Zonula Occludens-1
